# The Mechanical Interplay Between Differentiating Mesenchymal Stem Cells and Gelatin-Based Substrates Measured by Atomic Force Microscopy

**DOI:** 10.3389/fcell.2021.697525

**Published:** 2021-06-21

**Authors:** Hongxu Meng, Tina T. Chowdhury, Núria Gavara

**Affiliations:** ^1^School of Engineering and Materials Science, Queen Mary University of London, London, United Kingdom; ^2^Unit of Biophysics and Bioengineering, Medical School, University of Barcelona, Barcelona, Spain

**Keywords:** stem cells, AFM, biomechanics, biomaterial, osteogenesis

## Abstract

Traditional methods to assess hMSCs differentiation typically require long-term culture until cells show marked expression of histological markers such as lipid accumulation inside the cytoplasm or mineral deposition onto the surrounding matrix. In parallel, stem cell differentiation has been shown to involve the reorganization of the cell’s cytoskeleton shortly after differentiation induced by soluble factors. Given the cytoskeleton’s role in determining the mechanical properties of adherent cells, the mechanical characterization of stem cells could thus be a potential tool to assess cellular commitment at much earlier time points. In this study, we measured the mechanical properties of hMSCs cultured on soft gelatin-based hydrogels at multiple time points after differentiation induction toward adipogenic or osteogenic lineages. Our results show that the mechanical properties of cells (stiffness and viscosity) and the organization of the actin cytoskeleton are highly correlated with lineage commitment. Most importantly, we also found that the mechanical properties and the topography of the gelatin substrate in the vicinity of the cells are also altered as differentiation progresses toward the osteogenic lineage, but not on the adipogenic case. Together, these results confirm the biophysical changes associated with stem cell differentiation and suggest a mechanical interplay between the differentiating stem cells and their surrounding extracellular matrix.

## Introduction

Mesenchymal stem cells (MSCs) lineage specification is tightly regulated by their microenvironment through a wide variety of chemical and mechanical cues (Mao et al., 2015). Since the seminal work of Engler et al. (2006), showing that the stiffness of the cell’s substrate had a strong influence on stem cell differentiation, a growing variety of biomaterials have been engineered to manipulate stem cell behavior (Kfoury and Scadden, 2015; Li et al., 2016; Dalby et al., 2018). In particular, gelatin, a derivative of collagen, is considered an optimal biomimetic scaffold, especially to promote cell adhesion and development (Huebsch et al., 2010; Rustad et al., 2012). Since scaffolds made of pure gelatin are fragile, they are often reinforced with crosslinkers (Chaudhuri et al., 2016) such as genipin (Zhang et al., 2019). Previous studies have demonstrated the broad applications in which genipin-crosslinked gelatin hydrogel can be used, ranging from nerve and skeletal muscle regeneration to cartilage and bone repair (Gattazzo et al., 2018). Further studies have confirmed the ability of embedded cells to slowly reorganize, modify or degrade this type of hydrogel as part of bone repair mechanisms (Annamalai et al., 2018; Sung et al., 2018) thus showing their potential and biocompatibility in tissue regeneration.

The mechanical properties of a cell can reflect cellular state and are closely related to diseases and pathological conditions (Fletcher and Mullins, 2010). The cytoskeleton, a dynamic network of interconnected filamentous polymers and associated proteins regulates the mechanical properties of eukaryotic cells (Harris et al., 2019). A growing body of research suggests the readiness and accuracy (Engler et al., 2006) of adopting cell mechanics and cytoskeleton organization parameters for identifying cell phenotypes as biomarkers for indicating disease state (Rianna and Radmacher, 2016), aging (Lee et al., 2007), and development (Fernandez-Gonzalez and Zallen, 2009) at single cell level. In the particular case of stem cell differentiation, a number of studies have shown that their mechanical properties are largely influenced by the mechanical properties of their surrounding extracellular matrix (ECM) (Reilly and Engler, 2010). For example, cells plated on stiffer substrates became stiffer themselves (Kim et al., 2020) and the use of hydrogels with reversible mechanics has shown hMSCs responded dynamically to the reversible mechanical signaling (Rosales et al., 2017). While promising, these studies measured the cell mechanical properties only at an early stage of differentiation and it remains unclear how the mechanical properties of stem cells change throughout their differentiation (Hecht et al., 2015). Finally, recent work has linked cell-induced degradability of bioscaffolds with enhanced differentiation toward bone cell precursors (Khetan et al., 2013), promoting the use of bio-mimetic scaffolds that can be degraded or modified by the cells cultured onto them (Yao et al., 2017; Cristofaro et al., 2018). The question thus arises on whether a bi-directional mechanical interplay may exist between hMSCs and ECM during the course of differentiation, so that the mechanical properties of the bioscaffold change alongside those of the cells cultured on them.

We have previously shown that the cell’s mechanical properties and the underlying organization of the cell’s cytoskeleton are strongly correlated and that they can be used to better understand at the cellular level physiological processes such as aging or cancer (Gavara, 2016; Gavara and Chadwick, 2016). In particular, we have used Atomic Force microscopy (AFM) in the past to carry out cell indentation experiments and report mechanical properties of cells such as their stiffness (measured as Young’s modulus), viscosity and adhesion (Sliogeryte and Gavara, 2019; Zhang et al., 2020). Moreover, recently proposed contact mechanics models for cells adherent on soft substrates (Rheinlaender et al., 2020) or novel approaches for advanced data analysis of force-indentation curves (Keeling and Gavara, 2020) have allowed us to measure via AFM-indentation in a non-artifactual way any region of an adherent cell and also split the contributions of cortical and cytoskeletal stiffness. Of note, the recent introduction of force-feedback modalities in commercial AFMs allows mapping not only the cell topography, but also simultaneously all the mechanical properties listed above. Therefore, by performing force-feedback imaging on sparsely cultured cells and then using the topography map to threshold the obtained maps based on height, we can not only measure mechanical properties of the cells, but also simultaneously those of their neighboring ECM (Laly et al., 2021). Put together, the convergence of recent advances in AFM operating modalities, contact mechanics modeling and force-indentation data analysis allows us for the first time to develop a robust multiparametric approach to characterize the mechanical changes of differentiating cells and their underlying ECM.

Using this approach, we have tracked at the single cell level the mechanical and morphological changes associated with hMSCs differentiation. In particular, the strongest trends found were a persistent increase in cytoskeletal stiffness for cells differentiating toward osteogenic linages and a consistent decrease in cytoskeletal stiffness for cells differentiating toward adipogenic lineages. Further, we have confirmed that the observed mechanical changes are associated with a marked reorganization of the underlying actin cytoskeleton. Finally, we have found that the differentiation process toward osteogenic lineages is also accompanied by mechanical changes in the neighboring gelatin scaffold. Our results thus point toward a mechanical interplay between differentiating cells and their ECM, and highlight the advantage of using biomimetic cell-modifiable soft substrates as scaffolds for regenerative medicine.

## Materials and Methods

### Gelatin Hydrogels Synthesis

Gelatin hydrogels (Gel) were made of gelatin powder (G2500, Sigma-Aldrich, United States) dissolved in either 10mM/20mM/40mM genipin (Challenge Bioproducts, Taiwan) solution at a concentration of 3%/6%/9% (w/v). The mixture was kept at 40°C under moderate stirring until crosslinking was started, as indicated by the solution turning into a blue color. The polymer solution was cast in the mold and the obtained samples were left at room temperature for 24 h until polymerization was complete. The formed hydrogels were immersed in 70% ethanol solution to remove the excess genipin and sterilization. Then, the sterilized hydrogels were washed with PBS for 3 times and cultured in cell culture medium for 24 h in cell incubator before cell seeding.

### Cell Culture

Primary bone marrow MSCs were sourced from Promocell, Heidelberg, Germany. Expanded MSCs were used at passage 4. The basic media that was used to maintain cells during experiments and for proliferation was DMEM (Sigma- Aldrich, St. Louis, MO, United States) supplemented with 10% FBS (Sigma), 0.1% FGF-basic1 (PeproTech) and 1% penicillin-streptomycin (Sigma). Cells were seeded at a concentration of 4000 cells/cm^2^. Growth media was removed and replenished every 3 days.

For osteogenic induction media (ODM), DMEM (X-Pan) was supplemented with 50 μM ascorbic acid (Sigma), 100 nM dexamethasone (Sigma) and 10 mM β-Glycerophosphate. For adipogenic induction media (ADM), DMEM was supplemented with 500 μM IBMX(Sigma), 1 μM dexamethasone (Sigma), 100μM indomethacin, and 10μg/mL insulin (Sigma). In both cases, induction media was removed and replenished every 3 days.

### Histochemistry Analysis and Epifluorescence Microscopy Imaging

#### Oil Red O Staining for Intracellular Lipids

Oil Red O stock solution was prepared by adding 300 mg of Oil Red O (Sigma O0625) to 100ml of 99% isopropanol (0.3% w/v). To begin the histochemistry protocol, 6 mL of Oil Red O stock solution were mixed with 4 mL DI water. Cell samples were thrice rinsed in sterile PBS and fixed with 4% paraformaldehyde for 10 min. After being washed twice with DI water, 60% isopropanol was added and left to incubate for 5 min at RT. Subsequently, the Oil Red O working solution was added and incubated for 5 min at RT. Finally, the cell samples were rinsed repetitively with DI water until the solution became clear and left for storage. Imaging was carried out using phase contrast mode with an inverted microscope (Leica DMI4000B) with a 10× 0.5 NA objective lens and a CCD camera (Leica DFC300FX).

#### Alizarin Red Staining for Calcium Deposition

Samples were thrice rinsed in sterile PBS and fixed with 4% paraformaldehyde for 10 min. Following, the samples were rinsed with distilled water, and stained with 0.5 mL of Alizarin Red S solution (2 g/100 mL, pH = 4.1–4.3) for 10 min. Excess dye was rinsed with distilled water, and microscopy images were acquired by an inverted microscope (Leica DMI4000B) with a 10× 0.5 NA objective lens and a CCD camera (Leica DFC300FX).

#### Actin Cytoskeleton Visualization and Quantification of Its Organization

Mesenchymal stem cells cultured on gelatin hydrogels were fixed in 4% PFA (Sigma) and then permeabilized with 0.1% Triton X-100 (Sigma). Samples were incubated with Phalloidin CruzFluor^TM^ 488 Conjugate (Santa Cruz) at 1:500 dilution and 1:1000 DAPI (Santa Cruz) for 30 min at room temperature. All chemicals were purchased from Sigma Aldrich. Then samples were washed by PBS for 3 times and transferred with the seeded surfaces facing down into a cover glass. Zeiss LSM710 Confocal Microscope (Germany) was used to image cytoskeleton at 20× magnification and a cooled CCD camera.

The cytoskeleton organization parameters were analyzed according to our previous studies using a dedicated pipeline (CSKMorphometrics) written in MATLAB (Sliogeryte and Gavara, 2019; Zhang et al., 2020).

### Mechanical Characterization by AFM

The mechanical properties of MSCs cultured on gelatin gels were assessed via AFM using QI mode (Nanowizard 4, JPK Instruments AG, Berlin, Germany). To probe the samples, a pyramidal silicon nitride tip, with a cantilever spring constant of ∼0.03 N/m (MSNL-10D, Bruker) was used. Measurements were carried out by performing force-indentation ramps, using 100 × 100 μm range at a 2 μm/s ramping speed. A grid of 32 × 32 pixels was generated by performing a force-indentation curve for each pixel. The grid was defined broad enough to include also the surface of the gel neighboring the probed cell. Therefore, for each obtained grid, approximately half of the probed pixels corresponded to the cell and the other half to the surface of the gelatin gel. A minimum of 10 cells were probed for each timepoint and condition.

During the data post-processing steps, the pixels on each grid were classified and split as belonging to the cell or the gelatin substrate based on their measured height, using Otsu’s method for image thresholding (Laly et al., 2021). Subsequent force-indentation curve analysis was dependent on whether a given pixel had been classified as “gel” or “cell.” For “gel” pixels, the force-indentation curves were analyzed using Bilodeau’s model for a conical indenter. Similarly, viscosity was obtained using the model proposed by Radmacher’s group (Rebelo et al., 2013). For “cell” pixels, the force-indentation curves were again analyzed using Bilodeau’s model for a conical indenter and corrected using the CoCS model (Rheinlaender et al., 2020) for cells cultured on soft substrates. We further characterized the mechanical properties of both the cell cortex (cortical stiffness) and cytoskeletal stiffness. To do so, for each obtained force-identation curve we first identified the contact point and then we fitted only indentations between 150 and 400 nm (cortical), or indentations of 750 nm and onward (cytoskeletal), imposing the initially found contact point into the model (Keeling and Gavara, 2020). Viscosity was computed using the same approach described above for “gel” pixels. All force-curve analysis was performed using custom-written MATLAB codes.

### Quantitative Polymerase Chain Reaction

Cells were seeded in 35mm-diameter petri dish with seeding density of 30000 cells per dish and cultured for up to 14 days as described above. RNA was extracted using the RNeasy Plus Mini Kit (Qiagen, 73404) under manufacturer’s instructions at day 1, 7, and 14, and first-strand cDNA synthesized using QuantiTect Rev.Transcription Kit (Qiagen, 205311). Quantitative PCR was performed using the Taqman single-gene expression assay (Thermo Fisher Scientific) in a QuantStudio 7 Flex System (Thermo Fisher Scientific). The following oligonucleotide primers were used: PPARA (Hs00231882_m1) for adipogenesis and RUNX2 (Hs00231692_m1) for osteogenesis. In addition, GAPDH (Hs02758991_g1) was used for normalization. The comparative Ct method was applied to give relative gene expression values. All the values were compared with that gene expression at day 1.

### Statistical Analysis

Statistical analysis was performed in GraphPad Prism. Each experiment was performed independently in triplicate. Data are presented as mean ± standard deviation (SD) values or mean ± standard errors (SE) according to different situations. For the majority of analysis, nested 1-way ANOVA followed by multiple comparisons against “day 1” data using Dunnett’s test was used. A *P*-value of < 0.05 was considered significant.

## Results

### Gelatin Hydrogel Crosslinked by Genipin Has a Suitable Stiffness for Long-Term hMSCs Study

**FIGURE 1:**
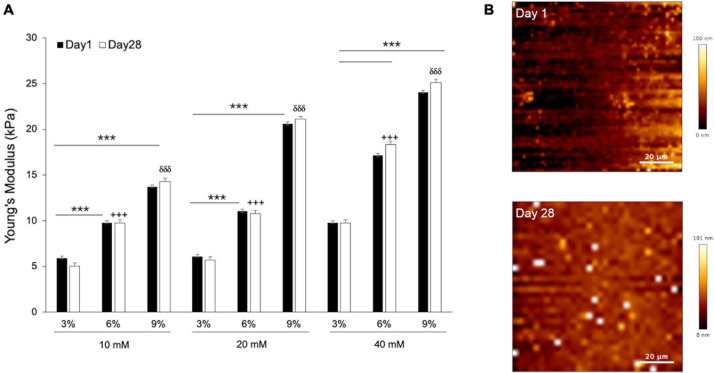
The mechanical properties and topography of hydrogels scale with gelatin and genepin concentrations. **(A)** Gelatin hydrogels from 3, 6, and 9% (w/v) were crosslinked with 10, 20, and 40 mM genipin solution and cultured for up to 28 days. All values for Young’s Modulus were compared to controls (Day 1). Error bars represent SD for *n* = 18 replicates from three separate experiments, where comparisons between 3% vs. 6% are indicated with a cross (+ + +) and 3% vs. 9% with a delta (δδδ). **(B)** Surface of gelatin substrates scanned by AFM at Day 1 and Day 28. Student-test comparisons revealed ***P < 0.001. All other comparisons were not significant.

In order to find the composition of gelatin-genepin gels for a given optimal stiffness (10 kPa), we produced a panel of 3%/6%/9% (w/v) gelatin hydrogels crosslinked in 10mM/20mM/40mM genipin solution. Subsequently, we measured their stiffness (reported throughout the manuscript as Young’s modulus) at 37°C using AFM. As expected, we observed a linear increase in hydrogel stiffness with increased gelatin powder concentration. For the 9% gelatin hydrogels, their stiffness grew linearly with increasing genipin solution. Conversely, for the 3% and 6% gelatin hydrogels, their stiffness did not significantly change until the concentration of genipin solution reached 40mM (Figure 1A).

Following AFM measurements, the hydrogels were sterilized and immersed in basic medium, and subsequently kept inside a cell culture incubator for 28 days. This procedure was carried out to confirm the stability of their mechanical properties when kept for a long time in temperature and bathing conditions that mimicked those of cell culture. After 28 days of incubation, the stiffness of all genipin-crosslinked gelatin hydrogels displayed no significant differences, thus confirming their mechanical stability in cell-less physiological conditions (Figure 1A). Simultaneously, the surface geometry of gelatin hydrogels was measured at both time points. As can be seen from Figure 1B, the height range of surface fluctuation was less than 100 nm and can therefore be regarded as flat at the cellular level.

For all subsequent experiments using genepin-gelatin hydrogels, we chose to use only those composed of 6% gelatin in 10mM genipin solution. The reasoning was that the obtained stiffness (∼10 kPa) falls between those reported for adipogenic tissue (at around 4 kPa) and the bone tissue (at around 30 kPa) (Discher et al., 2009), while requiring the minimum dosages of genepin. For the remaining of the study, we thus used these hydrogels as soft biomimetic substrates. First, we performed biocompatibility tests of our hydrogel model by using a CCK8 kit. The results (Supplementary Figure 1) confirmed that gelatin hydrogels crosslinked with genipin were non-toxic in cell culture conditions and could be hereby used as hMSCs substrates.

### Histological Staining and Gene Expression Profiles of MSCs Validated the Differentiation Processes During Osteogenesis and Adipogenesis

**FIGURE 2:**
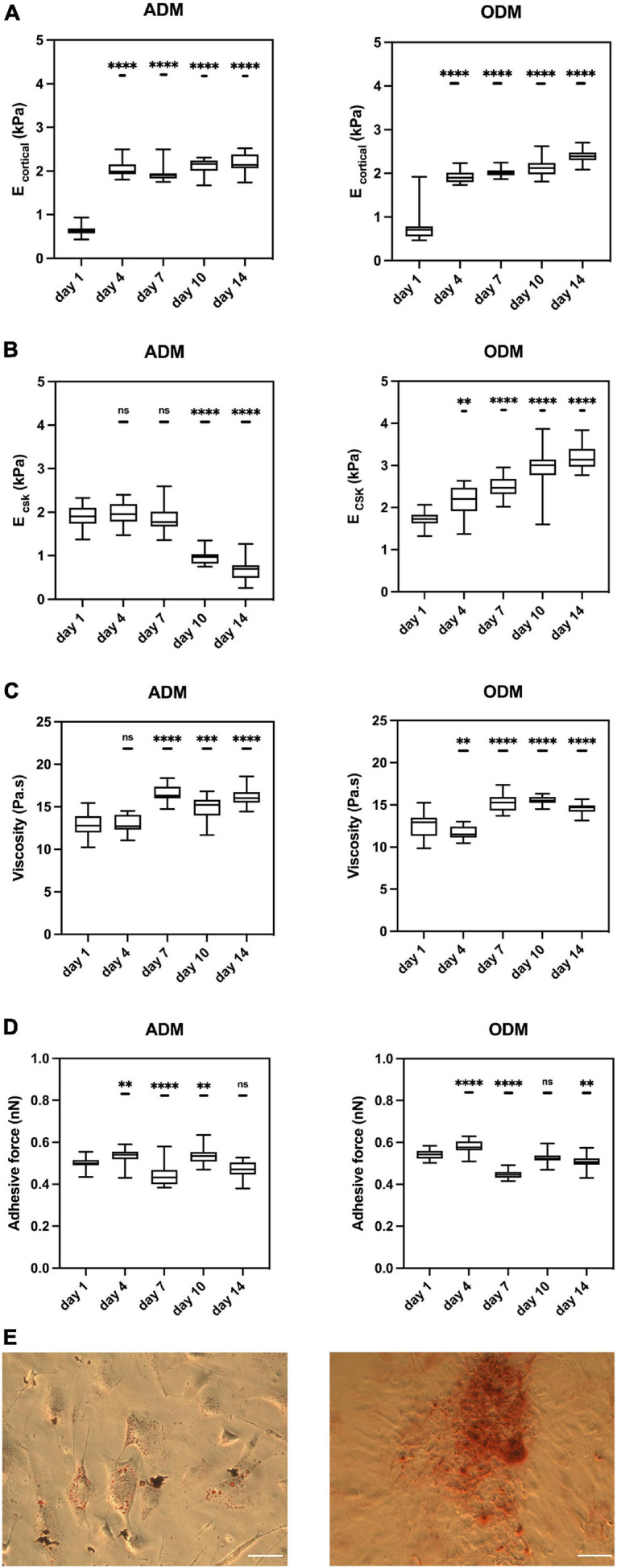
The mechanical properties of hMSCs change during differentiation. Box plots show the results of the **(A)** E _cortical_, **(B)** E _CSK_, **(C)** viscosity, and **(D)** adhesive force of hMSCs during adipogenic/osteogenic differentiation at different time points. **(E)** Red oil staining (left) for ADM and Alizarin red staining (right) for ODM at Day 14. Scale bar = 1mm. Box plots extend from the 10th to the 90th percentile, whiskers from min to max. A total of *n* = 71 (day 1, ADM), *n* = 69 (day 4, ADM), *n* = 75 (day 7, ADM), *n* = 56 (day 10, ADM), *n* = 54 (day 14, ADM), *n* = 71 (day 1, ODM), *n* = 69 (day 4, ODM), *n* = 85 (day 7, ODM), *n* = 73 (day 10, ODM), *n* = 67 (day 14, ODM), and cells were analyzed from *n* = 4 repeats. Asterisks indicate a statistical difference (^∗∗^*P* < 0.01, ^∗∗∗^*P* < 0.001, ^****^P < 0.0001, and obtained using a nested 1-way ANOVA design followed by a multiple comparisons Dunnett’s test against day 1). ns indicates not significant comparison.

hMSCs committed to different cell specifications on gelatin hydrogel, as confirmed by histological staining methods at Day 14. As expected, the presence of lipid droplets was observed in hMSCs cultured in ADM and phosphate deposits were found in hMSCs cultured in ODM (Figure 2E). Similarly, the gene expression of differentiating hMSCs was measured at days 1, 7, and 14 using standard differentiation markers. For cells cultured on ADM, we measured the expression levels of PPARA, which is considered to be a master adipogenic regulator (Zubiría et al., 2020). PPARA levels in ADM-cultured hMSCs kept increasing and reached a fourfold increase at day 14 (Supplementary Figure 2A). For cells cultured on ODM, we measured the expression levels of RUNX2, which is considered an early marker for osteogenesis and is typically observed to decrease in more mature osteogenic hMSCs (Tsimbouri et al., 2017). In our experiments in ODM cultured hMSCs, RUNX2 expression increased at day 7 indicating that osteogenesis had been initiated, and decreased at day 14, suggesting a more mature state had been achieved (Supplementary Figure 2B).

### The Mechanical Properties of hMSCs Undergo Significant Changes for Distinct Cell Line Specifications

Single cell topography and morphology were carried out by AFM at different time points. For both groups, cell shape and height were similar until Day 7 (not shown). As a qualitative observation, we found that hMSCs in ADM increased in height, likely as lipids accumulated in their cytosol, while cells in ODM started to remodel their neighboring ECM, which became rougher in its topography.

**FIGURE 3:**
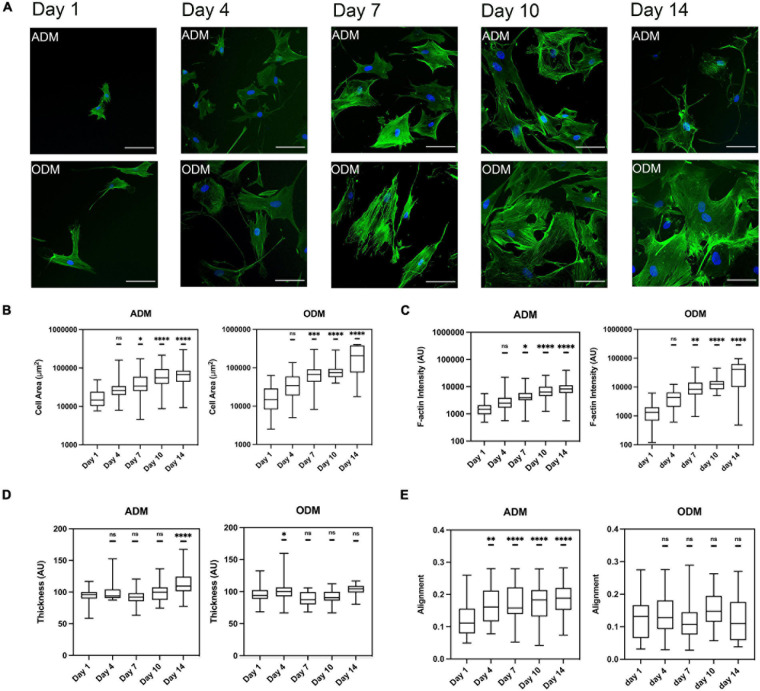
The F-actin cytoskeleton change of hMSCs during differentiation. **(A)** Cytoskeleton of hMSCs in ADM (1st row) and ODM (2nd row) at different time points, blue for DAPI and green for phalloidin, scale bar 100 um. Box plots show the results of the **(B)** cell area, **(C)** F-actin fiber intensity, **(D)** thickness, and **(E)** alignment of hMSCs during adipogenic/osteogenic differentiation at different time points. Box plots extend from the 10th to the 90th percentile, whiskers from min to max. A total of *n* = 45 (day 1, ADM), *n* = 25 (day 4, ADM), *n* = 40 (day 7, ADM), *n* = 52 (day 10, ADM), *n* = 39 (day 14, ADM), *n* = 44 (day 1, ODM), *n* = 66 (day 4, ODM), *n* = 26 (day 7, ODM), *n* = 19 (day 10, ODM), *n* = 13 (day 14, ODM), and cells were analyzed from *n* = 3 repeats. Asterisks indicate a statistical difference (^∗^*P* < 0.05, ^∗∗^*P* < 0.01, ^∗∗∗^*P* < 0.001, ^****^P < 0.0001, and obtained using a nested 1-way ANOVA design followed by a multiple comparisons Dunnett’s test against day 1). ns indicates not significant comparison.

We measured the mechanical properties of hMSCs at days 1, 4, 7, 10, and 14. At day 1, the mechanical properties of hMSCs cultured in either ADM or ODM were still very similar, and in both cases the stiffness of the cortical actin layer (around 0.5 kPa) was much lower than the stiffness of the cytoskeletal fiber’s underneath (around 2 kPa). For hMSCs cultured in ADM, the cortical stiffness had quadrupled to around 2 kPa by day 4 but remained constant for all later time points. This observation suggests that the re-organization of cortical actin takes place at an early stage of differentiation, with no further changes happening after that. Conversely, the cytoskeletal stiffness of ADM cells fell slightly between days 1 to 7 and then dropped markedly to around 0.5 kPa at day 14. Remarkably, the cytoskeletal stiffness observed at day 14 displays a similar stiffness to those reported for fat tissues at around 0.3 kPa (Samani et al., 2003). In addition, the temporal changes in hMSCs cytoskeletal stiffness were correlated with their differentiation status as the adipogenesis is reported to be observable after 1 week. As can be seen from Figure 3, the cytoskeleton of hMSCs in ADM was greatly altered at day 10. Of note, actin-less circular areas were observed in cells, likely corresponding to lipid vesicles. This phenomenon became more obvious at day 14, with the clear appearance of grape-like clusters (Figure 3). Simultaneously with the appearance of these actin-less clusters, cytoskeletal stiffness values were observed to decrease (Figure 2B).

For hMSCs cultured in ODM, both the stiffness of the cortical actin layer and the underlying cytoskeleton increased monotonically with time, reaching at day 14 values that were fourfold and twofold those of day 1, respectively. In addition, and similar to the observations in ADM cells, the principal changes in cortical actin stiffness took place at the earliest time points, followed by a subsequent stabilization of their values. Conversely, the values of cytoskeletal stiffness displayed a rate of increase that was smaller but prevalent in time.

The values for cellular viscosity and adhesive force were also obtained through the acquisition of AFM force-indentation curves. For both ADM and ODM, the values of viscosity increased significantly with time, even though the trends were less marked than those displayed for elasticity (Figure 2C). Similarly, no clear trends were observed for the values of adhesive force for either ADM or ODM differentiation (Figure 2D).

### Cytoskeleton of hMSCs Changes in Different Cell Line Specification

To obtain quantitative data cell gross morphology and F-actin organization, fluorescence images using phalloidin staining were obtained and subsequently processed using our image quantification pipeline. For both types of differentiation media used, cell area and F-actin assembly increased with time, even though hMSCs in ODM spread further since day 7 (Figures 3B,C). Both the value of cell area and F-actin assembly of ODM cells doubled those of ADM cells at day 7, and the gap widen further to threefold (cell area) and fourfold (f-actin assembly) at day 14. On the contrary, the organization of f-actin fibers displayed distinct organization for ADM and ODM. In particular, fiber alignment of ADM increased (fibers distributed in increasingly random directions) while those of ODM remained preferentially organized in parallel directions (Figure 3E). Finally, the thickness of f-actin fibers in the ODM cells didn’t markedly change but for ADM cells it slightly increased after day 7 (Figure 3D).

### The Gelatin Hydrogel Is Remodeled During Osteogenesis

**FIGURE 4:**
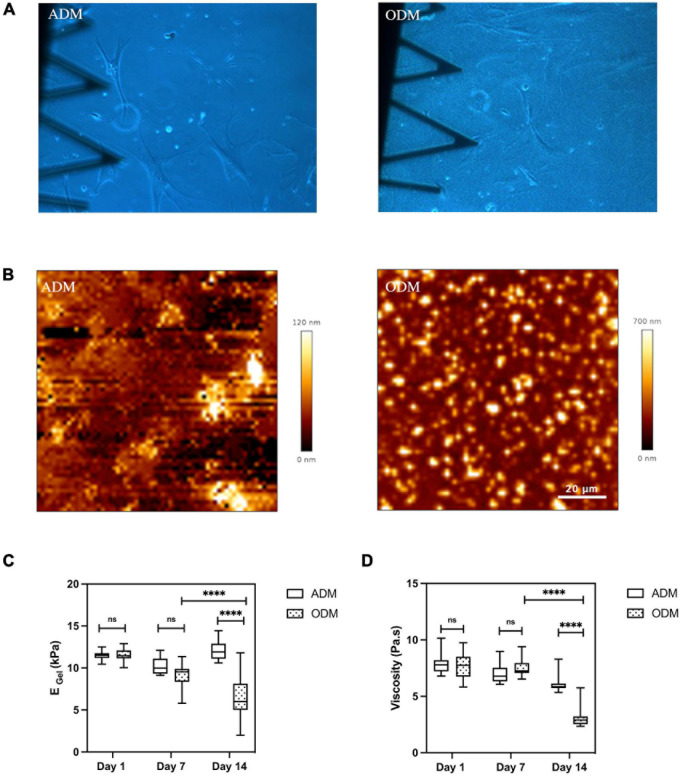
Gelatin hydrogel remolded during MSCs differentiation. **(A)** Optical images (10× magnification) of gelatin hydrogel at day 14. **(B)** Surface of gelatin substrates scanned by AFM at Day 14. Box plots show the results of the **(C)** Young’s modulus and **(D)** viscosity of gelatin hydrogel cultured with hMSCs in ADM/ODM at different time points. Box plots extend from the 10th to the 90th percentile, whiskers from min to max. Error bars represent SD for *n* = 6 replicates from three separate experiments. Asterisks indicate a statistical difference (^****^P < 0.0001, using a nested 1-way ANOVA design followed by a multiple comparisons Dunnett’s test against day 1). ns indicates not significant comparison.

In the present study, we were also able to monitor the changes in mechanical properties of the soft hydrogels in the vicinity of cultured cells. We found that the interaction between cell and gelatin scaffold could lead to matrix remodeling and measurable changes in the gels’ topography, stiffness, and viscosity. Matrix remodeling was already visible when assessing optical images obtained at low magnification using phase contrast imaging. For gels cultured with hMSCs in ODM, their surface became grainy, with small dot-like objects distributed randomly. In the particular case of a gel where ODM cells were kept for up to 9 weeks in culture, we even observed fibers over the gel’s surface (Supplementary Figure 3). Conversely, the gels appearance at the macroscale didn’t change visibly in the ADM condition (Figure 4A). Matrix remodeling in the case of ODM media cell culture was further confirmed at the nanoscle by AFM (Figure 4B), and the dimensions of the dot-like objects were measured to range from hundred nanometers to several micrometers in diameter, and up to 700 nm in height. Meanwhile, the topography of the gels cultured with hMSCs in ADM resembled that of freshly made gels (Figure 1B).

Through our force-indentation measurements, we were able to assess whether the changes in the gel’s topography in the case of ODM were also associated to changes in the mechanical properties. Indeed, we found that gel’s mechanics values showed a marked decrease with increasing time in ODM cell culture, dropping to 50% and 41% of their initial values by day 14 for Young’s modulus and viscosity, respectively (Figures 4C,D). Conversely, gelatin substrates maintained in adipogenic differentiation media retained their initial stiffness and viscosity (Figures 4C, D).

## Discussion

In this study, we have tracked at the single cell level the mechanical changes associated with hMSCs differentiation toward osteogenic and adipogenic lineages. While cortical actin stiffness displayed an abrupt and significant increase at the earliest time points of differentiation for both media, cytoskeletal stiffness showed significant and opposite monotonic changes between ODM and ADM for the duration of the experiments. The organization of the actin cytoskeleton was similarly significantly altered during differentiation, but the observed trends in ODM and ADM were largely parallel. Finally, we also found that ODM differentiation gave rise to mechanical softening and topography remodeling in the neighboring gelatin scaffold.

A number of previous studies have tracked the mechanical changes of stem cells upon differentiation induction using AFM (Liang et al., 2016; Zou et al., 2020). Nevertheless, the discrepancies between stem cell origin, induction media used, timeframe monitored and the specific AFM-indentation protocol used among them resulted in dissimilar findings between research groups, and make it difficult to reach overarching conclusions of the mechanical changes associated with stem cell differentiation. In spite of that, there is growing consensus that ODM differentiation leads to cell stiffening, while ADM shows either cellular softening or no marked changes in cell stiffness (Yu et al., 2010; González-Cruz et al., 2012; Labriola and Darling, 2015; Chen et al., 2016; Liang et al., 2016; Zou et al., 2020). In our study, we have followed an approach similar to that first presented by Sulchek’s group (Bongiorno et al., 2014; Islam et al., 2017), were a multiparametric single cell approach was used to monitor changes in cell morphology and correlate them with changes in cellular Young’s modulus and expression of bone-associated proteins such as osteocalcin and bone sialoprotein. In our case, we have focused our multiparametric approach on the mechanical characterization of single cells via mechanical maps obtained all along their spread area and advanced data analysis to compute additional mechanical parameters from each obtained force-indentation curve, thus providing the stiffness of the cortical actin layer and the underlying cytoskeletal stress fibers, in addition to cellular viscosity and adhesion. One of the key advantages of this approach is that our results are less impacted than those of others by the specific experimental protocol chosen. Previous studies have restricted the number of force-identation curves obtained per cell to less than 10, typically over the nucleus area, and have had limited control of the indentation depths used. Our approach deliberately uses very large indentations and hundreds of datapoints per cell during the experimental protocol, and then uses advanced data analysis approaches to split the results based on cellular depth and region alongs its spread area. As a result, we are able to identify cortical stiffness as the earliest mechanical indicator of differentiation onset. It should be noted that in our experiments cortical stiffness plateaus at day 4, and doesn’t display significant changes between ODM and ADM differentiation throughout the remaining of the experiment. Of note, AFM measurements on non-adherent ODM-differentiating cells also showed an early onset in cell stiffening followed by a plateau after day 1 (Bongiorno et al., 2018), thus suggesting that our cortical stiffness parameter probes the same actin structure as in AFM indentation experiments on non-adherent cells. Consequently, we suggest cortical stiffness is a useful mechanical parameter to assess early on that stem cells retain their ability to differentiate, but less so to distinguish between ODM and ADM differentiation. On the other hand, our measurements of cytoskeletal stiffness show a significant temporal increase for ODM versus a significant decrease for ADM, a behavior resembling that found when monitoring intracellular stiffness using video particle tracking micro rheology (Chen et al., 2016). Furthermore, the cytoskeletal stiffness values measured at day 14 are in line with those reported by others for cell stiffness of adipocytes and osteocytes (Bongiorno et al., 2014) thus indicating that the observed changes corresponded to cellular specialization along the expected linages. Consequently, we suggest that the consistent and opposing trends between ODM and ADM displayed by cytoskeletal stiffness during differentiation make it the most suitable parameter within out multiparametric approach to assess a successful differentiation process. Of note, in ADM the decreases in cytoskeletal stiffness appear alongside increases in cortical stiffness. This may give rise to confounding mechanical changes when using AFM probing to measure overall cell stiffness, if the indentation depths used are not fully controlled and consistent among probed cells or even among research groups. This effect may explain the discrepancies reported previously among different studies where different AFM probing protocols were used to monitor the changes of hMSC undergoing differentiation toward adipogenic lineages.

The organization of the actin cytoskeleton has also been considered a reliable biomarker of stem cell differentiation (Treiser et al., 2010), even though cellular stiffness changes may precede morphological changes (Bongiorno et al., 2018). In our case, we indeed find marked trends in cell gross morphology as well as the organization of actin fibers and their total amount as cells differentiate. In general, differentiating cells become more spread and accumulate f-actin fibers in a monotonic fashion. Of note, other morphological parameters that characterize the overall organization of actin fibers display non-monotonic changes, thus highlighting intermediate steps in the differentiation process where cells resemble neither a stem cell nor the fully differentiated counterparts (Bongiorno et al., 2018). It is worth stressing that even though we and others observed marked trends in cytoskeletal organization during differentiation, the behavior in cell spread area of f-actin amount for ODM and ADM is similar (Chen et al., 2016), even though the rate of increase for parameters such as cell spread area and F-actin amount being larger for ODM and ADM. As such, while cytoskeletal organization has been considered by others as a reliable biomarker of differentiation away from a stem cell-like state, its reliability is reduced when used to distinguish ODM versus ADM phenotypes (Chen et al., 2016). It should be noted that while our immunostaining experiments started with sparsely platted cells, confluency increased at the latest time points. As a result, the presence of neighboring cells is likely to affect the overall morphology of reported in our experiments. In this connection, it has already been suggested that confluence could negatively affect the reliability of actin organization as a biomarker in the particular case of ADM differentiation at late time points (Treiser et al., 2010).

It should be stressed that, even though it is single cell and multiparametric in nature, our methodological approach doesn’t allow us to track the same individual cells for days along their differentiation path, nor measure the exact same cells with all techniques presented here (AFM mechanical probing, immunostaining-based quantification of the cytoskeleton’s organization, histological staining and gene expression profiles). Because of that, we can’t rank our measured mechanical or CSK morphological parameters based on their positive (or negative) spearman correlation with day of differentiation of gene expression of lineage markers. Consequently, it is beyond the scope of this study to formally identify the most suitable biophysical biomarker of stem cell differentiation. Nevertheless, based only on the significances obtained in our statistical tests for all parameters measured, mechanical parameters appear to provide a more promising avenue than CSK morphological parameters, and as already discussed above, the cytoskeletal stiffness is the mechanical parameter that is more strongly related to differentiation lineage and maturity.

While the role of the cell’s substrate stiffness in directing stem cell differentiation has been known for more than a decade, fewer studies have focused on assessing the mechanical changes of stem cells as they differentiate when cultured on soft scaffolds. Here the consensus is that ODM differentiation leads again to cell stiffening and actin reorganization, and that the choice of substrate affects the magnitude and rate at which these changes take place (Topal et al., 2017; Yen et al., 2020). In this connection, synthetic materials such as polyacrylamide and PDMS are prevalently used as soft matrices for these type of differentiation studies. As such, it is not anticipated that differentiating stem cells will remodel this type of substrates. Of note, our study combines two less prevalent approaches. On the one hand, the use of biomimetic substrates that could be potentially remodeled by adherent cells (Annamalai et al., 2018; Sung et al., 2018). On the other hand, the ability to additionally monitor the mechanical properties of the neighboring ECM while we carry out AFM experiments on our probed cells. Together, this has allowed us to show that ODM differentiating cells remodel their neighboring ECM, in a process that involves ECM topographical reorganization and mechanical softening. Such cell-induced matrix remodeling is not observed in ADM differentiation. Importantly, a similar behavior was observed by others on stem cells cultured inside photopolymerized RGD-modified methacrylated hyaluronic acid hydrogels, where the authors showed that scaffold degradability was required for ODM differentiation (Khetan et al., 2013). Conversely, when cells where embedded into matrices that could not be degraded by cells, the population transitioned toward an ADM phenotype. Of note, in that study scaffold degradation in ODM differentiation was associated with increased intracellular tension of the cells, a phenomenon also reminiscent of the observed increases in cytoskeletal stiffness in our ODM differentiating cells. While all these constitute promising observations in scaffold mechanics, further work is still required to monitor whether they are associated with changes in the chemical composition of the neighboring ECM and pinpoint the specific roles of matrix degradation versus *de novo* deposition and synthesis. In any case, our results highlight that the use of degradable biomimetic scaffolds as soft substrates for stem cell differentiation should allow a broader characterization of the interplay between differentiating cells and their ECM, including a potentially dynamic and finely tuned mechanical interplay between cells and scaffold. Accordingly, we anticipate that the use of biomaterials that can be readily remodeled and mechanically altered by their harbored cells will promote and enhance the use of bioscaffold-based regenerative therapies.

## Data Availability Statement

The raw data supporting the conclusions of this article will be made available by the authors, without undue reservation.

## Author Contributions

HM and NG designed the studies. HM conducted all the experiments. HM, NG, and TC contributed to analyzing and interpreting the data, drafted the manuscript, and edited the final submission. All authors have read and agreed to the published version of the manuscript.

## Conflict of Interest

The authors declare that the research was conducted in the absence of any commercial or financial relationships that could be construed as a potential conflict of interest.
