# Supplementary material for: The Mechanical Interplay Between Differentiating Mesenchymal Stem Cells and Gelatin-Based Substrates Measured by Atomic Force Microscopy
Source: Front Cell Dev Biol. 2021 Jun 21;9:697525. doi: 10.3389/fcell.2021.697525 (PMC8255986; doi:10.3389/fcell.2021.697525)
Supplement: Supplementary file 1 [file Data_Sheet_1.docx]

Supplementary Material

Hongxu Meng^1^, Tina T Chowdhury^1^, Núria Gavara^1,2*^

1 School of Engineering and Materials Science, Queen Mary University of London, Mile End Road, London E1 4NS, UK

2 Unit of Biophysics and Bioengineering, Medical School, University of Barcelona, Casanova 143, 08036 Barcelona, Spain

* **Correspondence:**

Corresponding Author: Núria Gavara

ngavara@ub.edu

**Supplementary material**


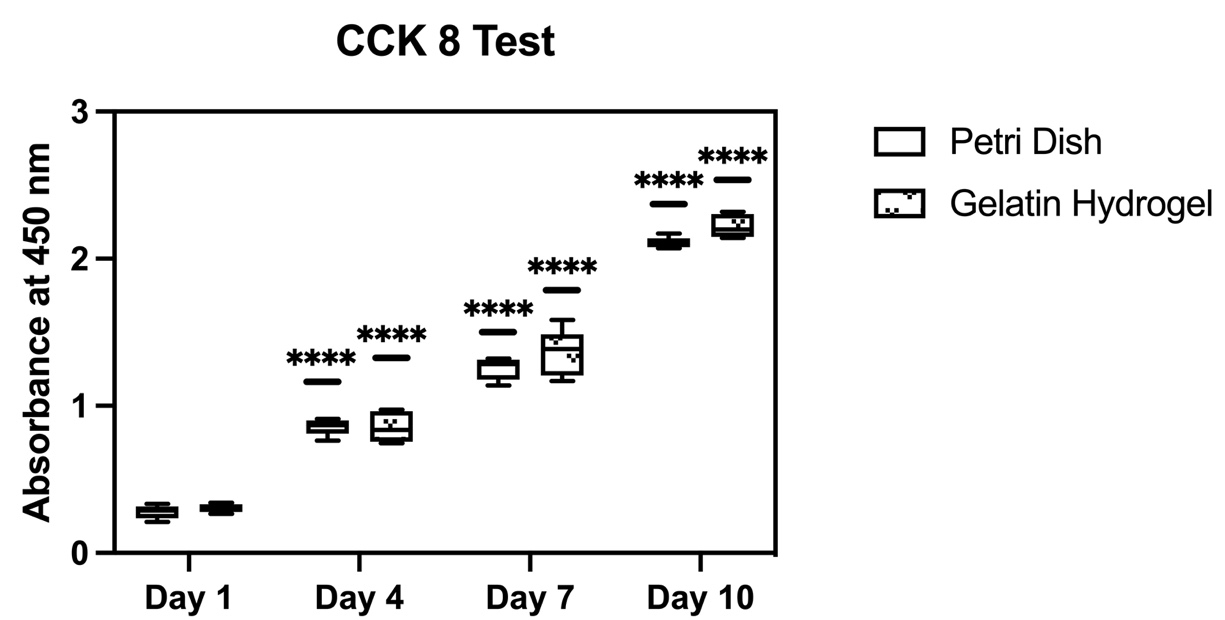


Supplementary Figure 1. hMSC proliferation on Petri dish and gelatin hydrogel evaluated by CCK-8 assay at different time points. Box plots extend from the 10th to the 90th percentile, whiskers from min to max. A total of n = 6 wells were analyzed. Asterisks indicate a statistical difference (*P < 0.05, **P < 0.01, ***P < 0.001, **** P < 0.0001, obtained using Dunnett’s test against day 1).


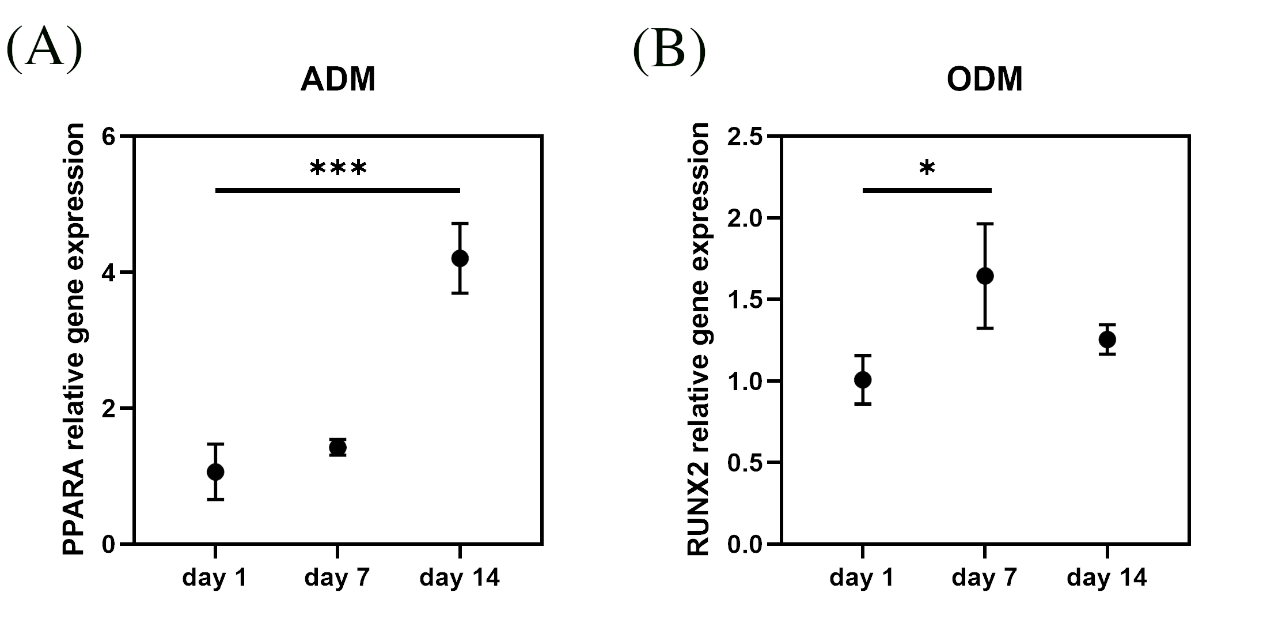


Supplementary Figure 2. Relative gene expression levels of standard differentiation markers. A) expression of adipogenic competency marker PPARA in hMSCs cultured in AD. B) expression of osteogenic early marker RUNX2 in hMSCs cultured in ODM. All values have been normalized to the average level found at day 1. * and *** indicate p<0.05 and p<0.001, respectively, as compared to day 1.


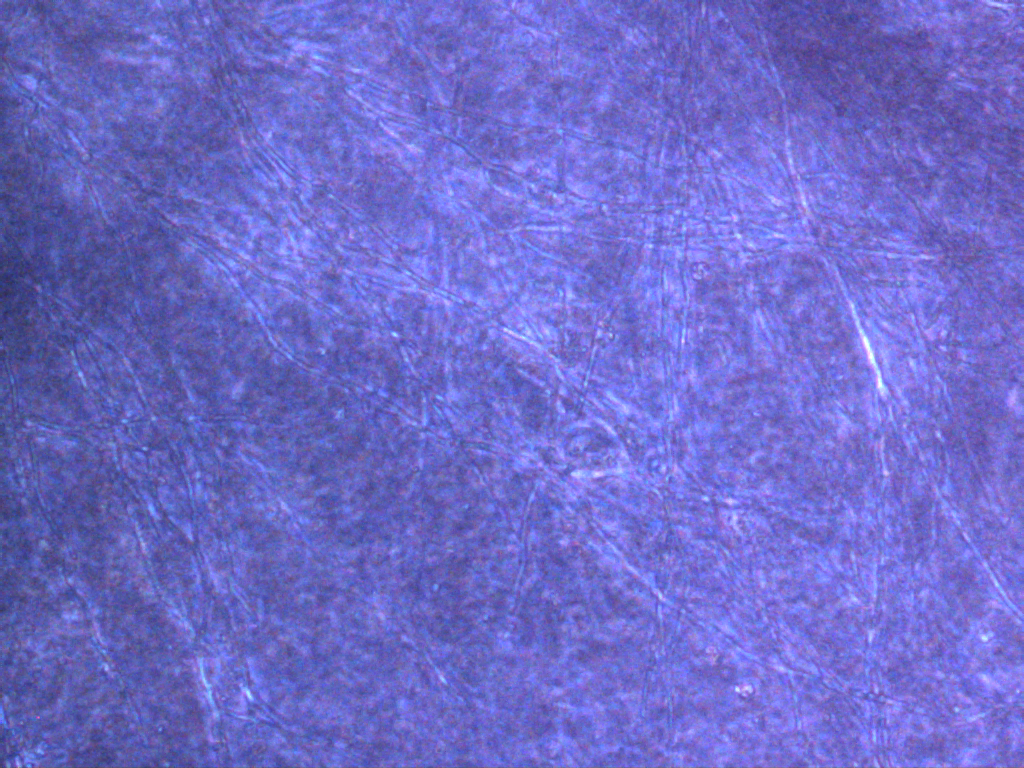


Supplementary Figure 3. Optical image (10x magnification) of a genepin-crosslinked gelatin hydrogel cultured with hMSCs in ODM for 9 weeks.
